# Thermal Behavior of Muscovite Sheet Mica

**DOI:** 10.6028/jres.067A.057

**Published:** 1963-12-01

**Authors:** Stanley Ruthberg

## Abstract

The three spectral types of muscovite sheet mica, i.e., very pink ruby, light green, and dark green, were subjected to heat treatments at temperatures up to 600 °C. The changes in the apparent optic axial angle and in the absorption spectra (0.3 to 15 *μ*) were studied along with color.

The differentiation of muscovite sheet according to these spectral types extends to the behavior of apparent optic axial angle and to certain regions of the spectrum under heat treatment. The pink associated absorption region (0.47 to 0.6 *μ*) can be enhanced or bleached away by appropriate thermal treatment, although the associated infrared multiplet at 3 to 3.5 *μ* is little affected. The absorption band at 12 *μ* increases in intensity with temperature of treatment. It is suspected that the 0.47 to 0.6 *μ* absorption is the result of color centers.

It has been shown that measurable differences exist in apparent optic axial angle and absorption spectrum, as well as in color, for muscovite sheet micas. These differences indicate that there must be basic chemical and structural variations. They further provide a quantitative, though complex, categorization of the material [1].^1^

The present paper reports the effect of heat treatment on color, apparent optic axial angle, and absorption spectrum for several of the representative categories of the material so established. The treatments were at temperatures of 600 °C and less, usually considered to be below the decomposition point.

## 1. Experimental Procedures

The details of measurement of apparent optic axial angle, absorption spectrum, and color are as before [1,2].

Comparisons of spectral variations are again made in terms of the resonance absorption coefficient (below background), *α*_λ_(*R*), defined with respect to an appropriately placed base line as

$$\alpha_{\lambda }\left(R\right)=-\frac{1}{t}\text{ln}\;\left(T_{R}|T_{b}\right)$$ (1)

where *T_R_* is the transmission at the resonance band center, *T_b_* is the value at the base line for the same wavelength, and *t* is the specimen thickness.

Color was determined by comparison with the previously selected standard samples.

### 1.1. Specimens

Three categories of muscovite sheet were chosen. These were very pink ruby, light green, and dark green, which represent the three end-types as defined in the earlier work.

Crystal sheets were all of high quality, V–1 to V–4 [3], with origins in India, Brazil, Tanganyika, and the United States.

As appropriate combination of visual color determination and restriction to magnitude of apparent optic axial angle can assure selection of these end-types, such procedure was used for sample choice. Specimens were first selected according to visual color. They were then picked for magnitude of apparent optic axial angle. The very pink ruby specimens were restricted to angles between 68° and 72.5°. Light greens were restricted to angles greater than 72.5°. Dark greens were taken with angles less than 68°. Selection was further restricted to those specimens for which repeatability was obtained in the measurement of angle to within 5′ of arc.

### 1.2. Heat Treatment

Specimens were air fired. Several temperature-time schedules were used which employed temperatures of 300, 500, and 600 °C and time intervals ranging from 3 min to 72 hr.

Samples were contained in covered Vycor crucibles to avoid contamination. Crucibles were first chemically cleaned and air fired at 500 °C. Clean procedures of sample handling were employed.

**Figure 1:**
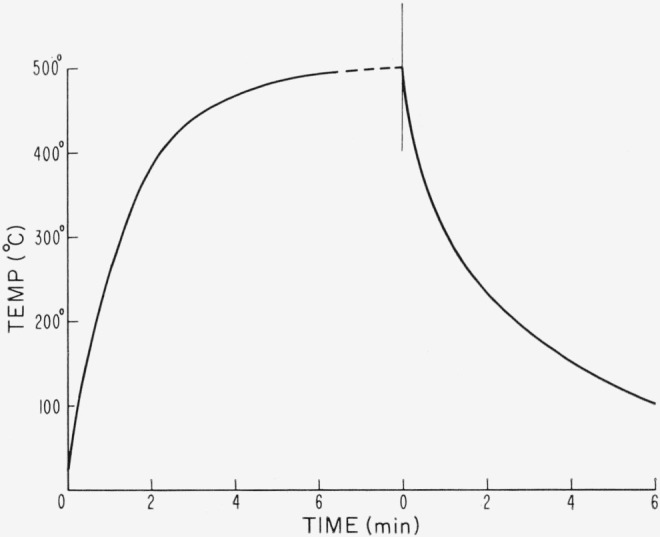
Heating and cooling curves for a representative sample subjected to a heating procedure at 500 °C. Crucible was thrust into furnace at 0 time on heating curve, and was extracted and removed from firebrick holder at 0 time on cooling curve.

To reduce thermal lag the firebrick supports for the crucibles were first brought to temperature in the muffle furnace. These were then quickly withdrawn, the loaded crucibles were set in, and the assembly moved back into the furnace. Withdrawal was in the reverse order. The interval of treatment was measured from the time when the crucible went into the furnace to that when it was withdrawn and taken out of the firebrick holder. The consequent heating and cooling data for a typical sample were as shown in figure 1. For these, temperature was measured with a fine thermocouple inserted between laminae. It is seen that 3 min in the furnace, set at 500 °C, caused the sample temperature to rise to ~430 °C and to be above 400 °C for about ½ min. Ten minutes in the furnace caused the sample temperature to be above 450 °C for over 6 min.

To lessen the number of runs, many samples were placed in each crucible. As a control procedure appropriate mixing of sample categories in these crucibles was used. In some runs samples from each end-type were mixed together. In other runs samples were grouped according to one type only.

Both sequential and individual temperature times were utilized, i.e., some samples were heated to a series of temperatures, for varying times, other samples received a single temperature-time treatment. Specific treatments were as follows:

1. Various time intervals at 300 and 500 °C, mixed samples—Crystal sheets were sectioned and distributed into a number of crucibles. Each crucible had three samples, one of each of the end-types, so placed within it as to be separated from each other, and each crucible received one particular temperature-time treatment. A temperature of 300 °C was used for time intervals of 90 min and 5, 24, 48, and 72 hr. A temperature of 500 °C was used for time intervals of 3, 10, 30, and 90 min, 5 and 24 hr.
2. Fixed interval of 24 hr at several temperatures, no mixing—Samples were sectioned and distributed into the crucibles so that only one type was in each crucible. Three crucibles, one for each type, were heated together for a specific time and temperature. The period of time was 24 hr. Temperatures were 300, 500, and 600 °C.
3. Interval of 90 min at 300 °C, no mixing—Same preparation as (2).
4. Successive treatments for 24 hr at several temperatures, mixed samples—Mixed types of single crystal sheets were heated successively at 300 °C–24 hr, then 500 °C–24 hr, and then 600 °C–24 hr.

When sectioned, a crystal sheet was cut into a number of pieces of small area not less than ½ in.×½ in. Angles and/or spectra were obtained before and after treatment on each individual section.

## 2. Results

### 2.1. Spectrum

The spectral changes due to heat treatment are substantial in the 0.3 to 1 *μ* region. The infrared region of 1 to 15 *μ* is less affected with little correlation found except in the 3 to 3.5 *μ* and 12 *μ* areas.

#### a. 0.3 to 1*μ*

**Figure 2:**
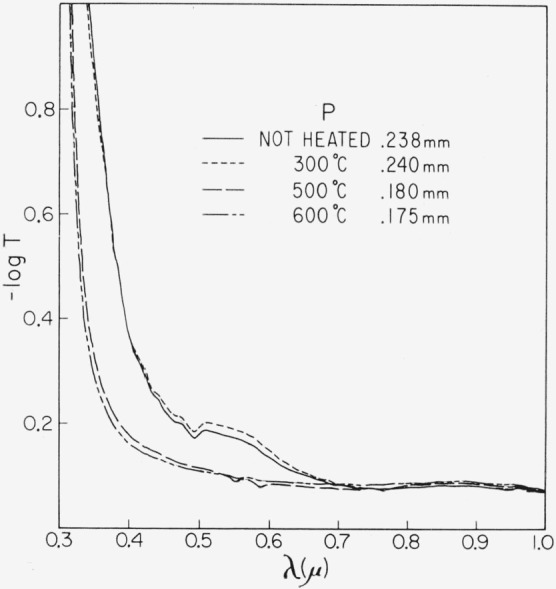
Change in the 0.3 to 1 μ absorption spectrum of a pink ruby with heating for 24 hr at various temperatures (No. 2 schedule). Specimen thickness is indicated.

**Figure 3:**
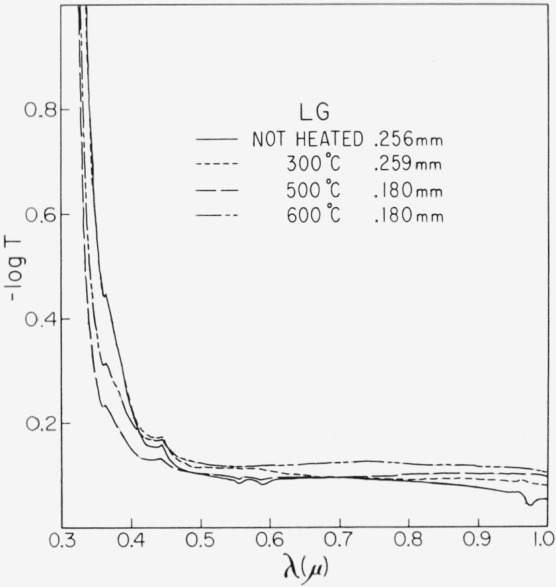
Change in the 0.3 to 1 μ absorption spectrum of a light green with heating for 24 hr at various temperatures (No. 2 schedule). Specimen thickness is indicated.

**Figure 4:**
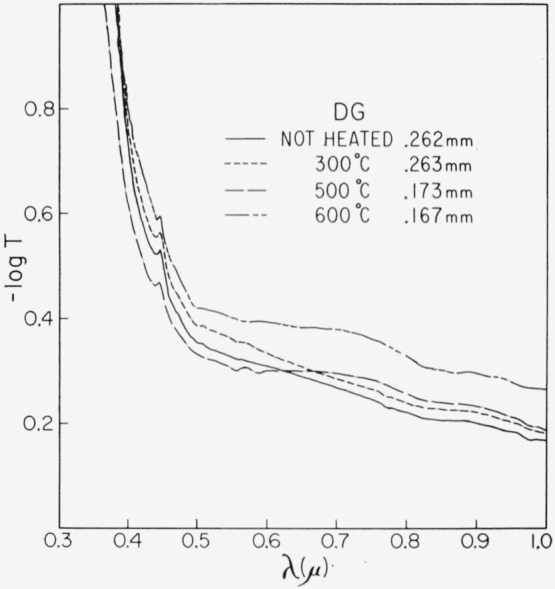
Change in the 0.3 to 1 μ absorption spectrum for a dark green with heating for 24 hr at various temperatures (No. 2 schedule). Specimen thickness is indicated.

**Figure 5:**
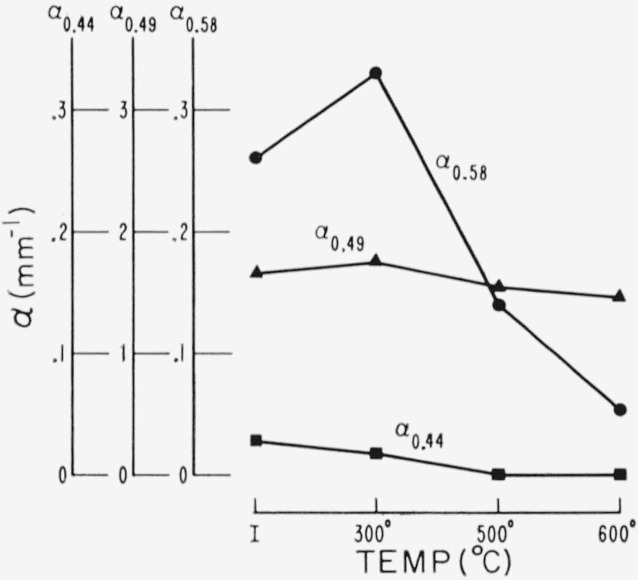
Change in visible spectrum of a pink ruby due to heating (No. 2 schedule) as described by absorption coefficients. α_0.44_ is green correlated. α_0.49_ is a measure of band edge absorption. α_0.58_ relates to visible pink associated absorption. I is initial untreated value.

**Figure 6:**
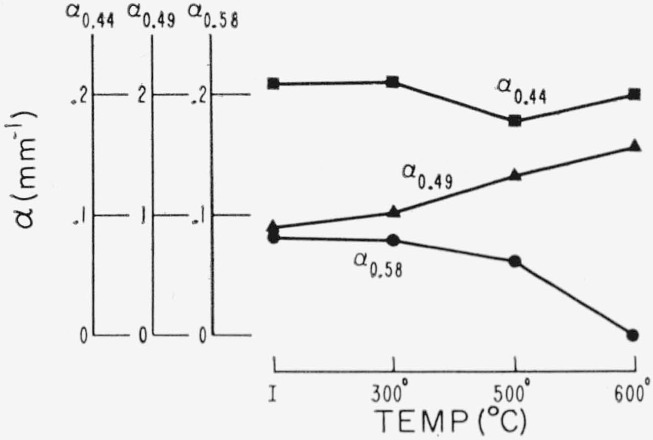
Visible spectrum change due to heating for a light green as described by absorption coefficients (No. 2 schedule).

**Figure 7:**
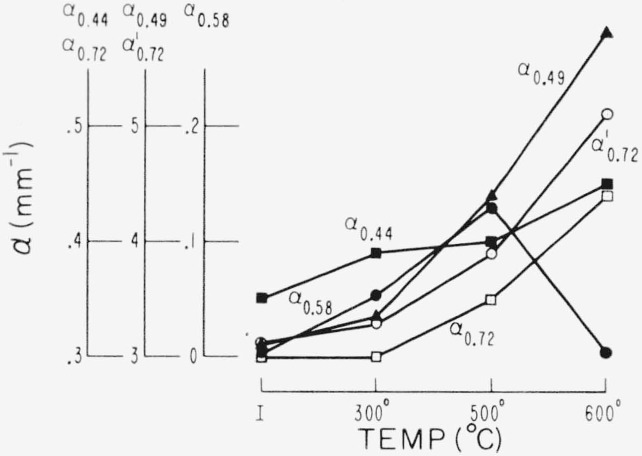
Visible spectrum change due to heating for a dark green as described by absorption coefficients (No. 2 schedule).

Representative results in the 0.3 to 1 *μ* region for the three end types are shown in figures 2, 3, and 4. Each figure is for a single crystal which had been cut into four sections. Each section received a specific temperature treatment for 24 hr (procedure No. 2). The very pink ruby and dark green types are most affected but with different overall rseponse. Quantitative results are given in figures 5, 6, and 7 in terms of the absorption coefficients for wavelengths of 0.44, 0.49, and 0.58 *μ*, which coefficients have been used previously to categorize muscovite sheet.

These data show that the red color of the pink ruby is enhanced by the low temperature (300 °C) treatment, but that bleaching results from the higher temperatures. The color changes from pink to a gray with residual pinkness after 500 °C and to gray after 600 °C. The deep absorption edge shifts to shorter wavelength. Transparency is also increased by 500 °C but tends to diminish after 600 °C.

The 0.47 to 0.6 *μ* pink associated absorption region is also enhanced in the dark green by 300 °C treatment, which caused the material to become khaki in appearance. The higher temperature again diminished the pink associated absorption and turned color to a very deep green. Opacity increased considerably and the absorption edge shifted strongly to longer wavelengths. Two additional coefficients have been introduced to describe the broad absorption which has appeared at ~0.72 *μ* as center wavelength. These are *α*′_0.72_, the total absorption coefficient at $0.72\;\mu \;\left(-\frac{1}{t}\text{ln}\;\;T\right)$ and *α*_0.72_, the resonance absorption coefficient, eq (1). These two coefficients measure height of the absorption as well as the base-to-peak value.

Light green was least affected. This typical specimen took on a slight gray cast after 300 °C treatment with a small increase in the pink associated absorption region at 0.47 to 0.6 *μ*; however, color returned to light green after the high-temperature treatments.

Pink ruby specimens which had been exposed to 500 °C for 24 hr were subsequently heated at 300 °C for as long as 52 hr. The pink associated absorption region of 0.47 to 0.6 *μ* was not restored. On the contrary, such treatment then caused a further leveling of the spectral profile. Bleaching does not appear to be spontaniously reversible. Induced visible changes have remained stable at room temperatures for over two years.

**Figure 8:**
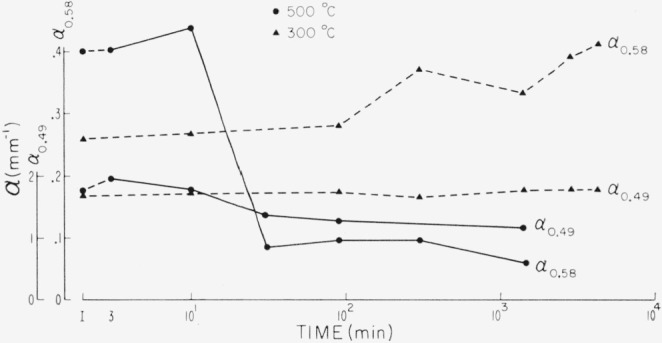
Effect of heating on the visible pink associated absorption region of 0.47 to 0.6 μ and on the height of the absorption edge of a very pink ruby. No. 1 procedure for heat treatment. α_0.58_ is a measure of the pink associated absorption. α_0.49_ is a measure of the height of the absorption edge and of transparency.

The response of the pink associated region is more complex than already shown. Typical kinetics of the reaction are shown in figure 8 for treatments at 300 and 500 °C. Whereas the red color is seen to increase with firing times at 300 °C, 500 °C treatment enhanced the red color after short treatment but bleached it away on longer exposures. At the same time opacity increased slightly with short exposure but diminished on long exposure.

**Figure 9:**
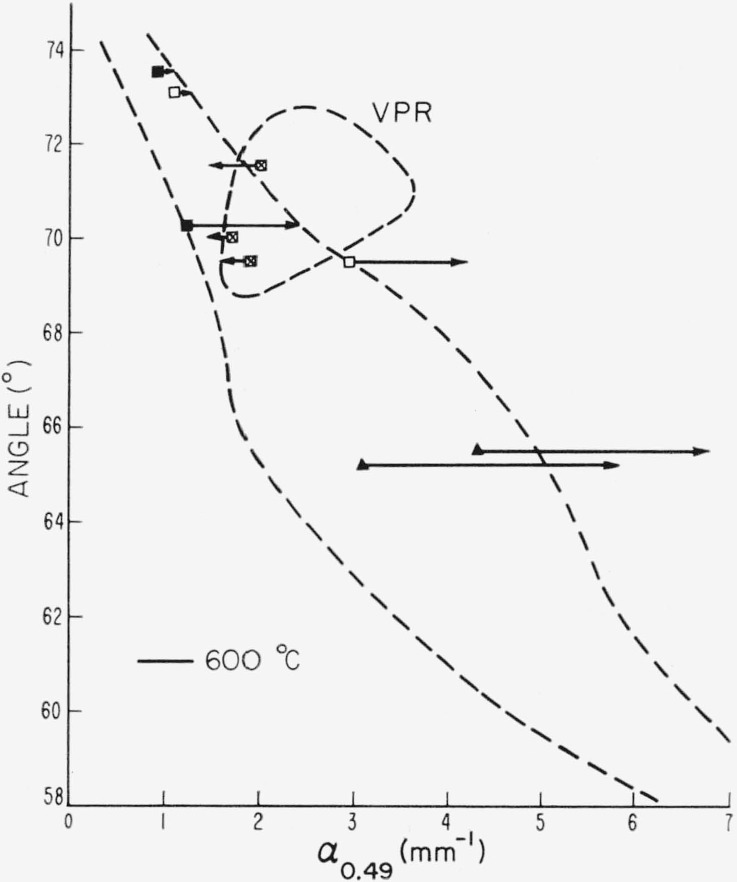
The magnitude and direction of change of α_0.49_ due to heating at 600 °C for 24 hr as a function of initial coordinates. ⊠—very pink ruby, □—ruby, ▲—dark green, ■—pale green.

The relationship of the shift in the absorption edge as a result of treatment at 600 °C to the initial value of apparent optic axial angle is shown in figure 9. The two outlined regions denote the general association previously found [1] for untreated samples between the height of the absorption edge, here measured by *α*_0.49_, and apparent optic axial angle. VPR designates the domain of very pink ruby specimens. The other region represents the general relationship for all specimens other than very pink ruby. The direction of change, as shown by the arrows, and the magnitude of change in *α*_0.49_, as shown by the length of the arrows, due to treatment are dependent upon the initial coordinates. Those specimens which originated within the VPR domain diminished in value of *α*_0.49_. All others increased in value.

#### b. 1 to 15*μ*

**Figure 10:**
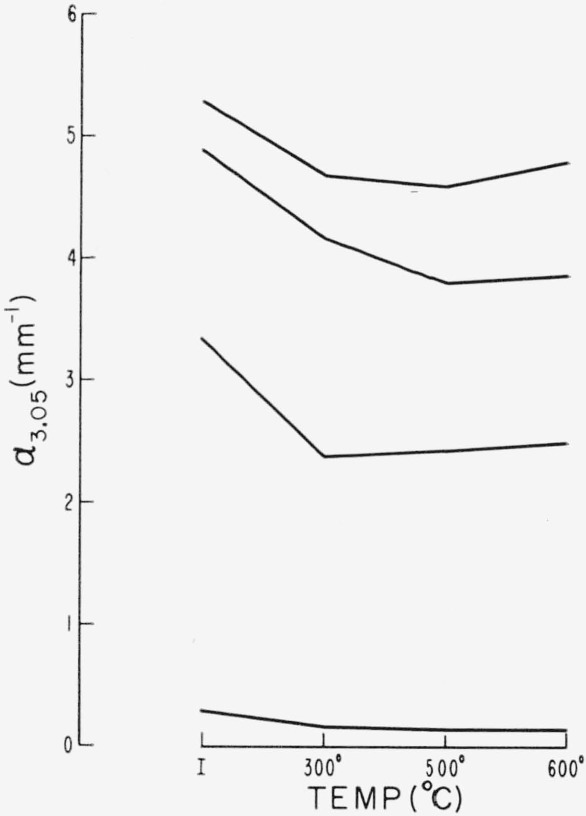
Effect of heat treatment on the infrared pink correlated structure as represented by α_3.05_ for various initial values. The upper three traces are for very pink specimens, while the lowest trace is for a green ruby [1].

The effect of treatment on the 3 to 3.5 *μ* pink correlated infrared region is shown in figure 10. Variations are shown for three very pink ruby specimens of different initial values of *α*_3.05_, and for comparison the results are included for a ruby specimen with small initial *α*_3.05_ (green ruby). Each crystal was subjected to sucessive temperatures of 300, 500, and 600 °C for 24 hr at each temperature (procedure 4). Although decreased by these treatments, the 3 to 3.5 *μ* absorption does not disappear. This is in contrast to the response of the visible pink associated region of 0.47 to 0.6 *μ.*

No noticeable increase in absorption was found in the 3 to 3.5 *μ* region in the light green and dark green types.

**Figure 11:**
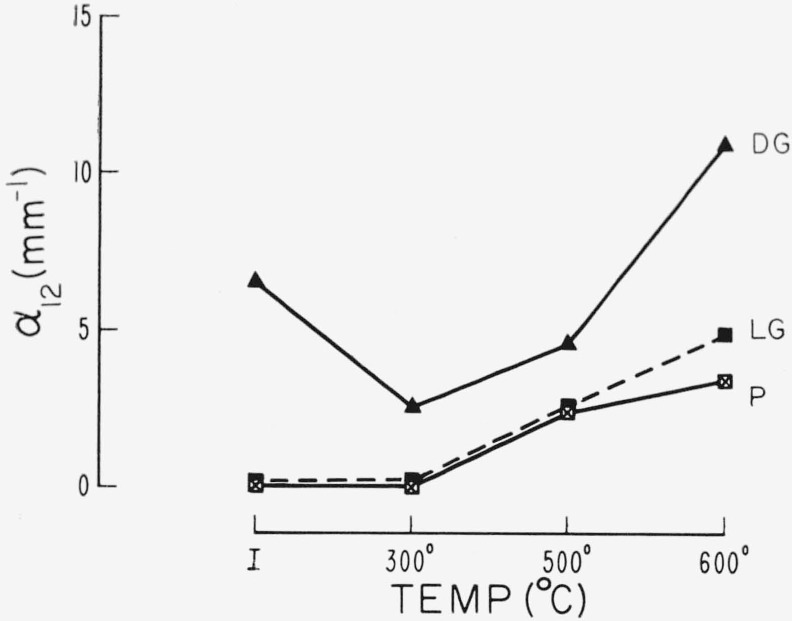
Change in α_12_ with heat treatment (No. 2 thermal schedule), for spectral types. Very pink ruby, light green, and dark green.

**Figure 12:**
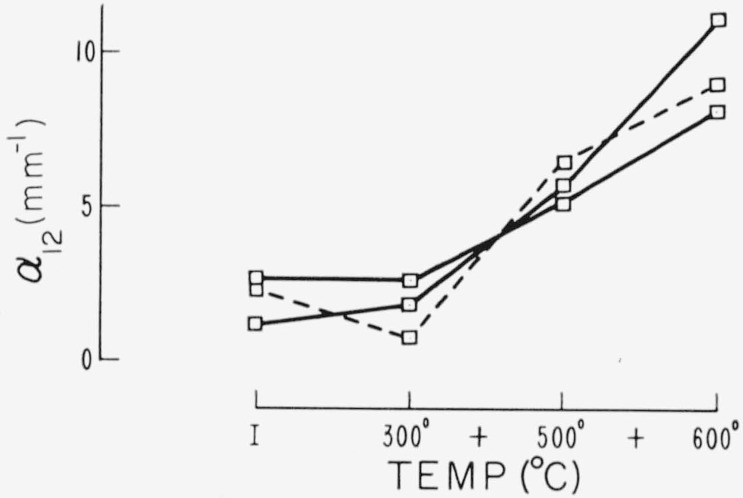
Effect of heat treatment on the 12 μ band of several ruby specimens (No. 4 procedure).

Changes in the 12 *μ* area are given in figures 11 and 12. In general heat treatment causes an increased absorption. Some specimens, as these examples show, decrease in absorption after low temperature heating. Such behavior is similar to that of the visible pink associated absorption; however, a consistent correlation of this effect with the color was not found.

No overall trends were found for the remaining bands of this region, although the thermal behavior of the 13.3 and 14.5 *μ* bands generally followed that of the 12.5 *μ* band, just as was the case for untreated specimens.

### 2.2. Apparent Optic Axial Angle

The apparent optic axial angle changes by fractions of a degree with these low temperature treatments and in a manner distinctive with each spectral type.

**Figure 13:**
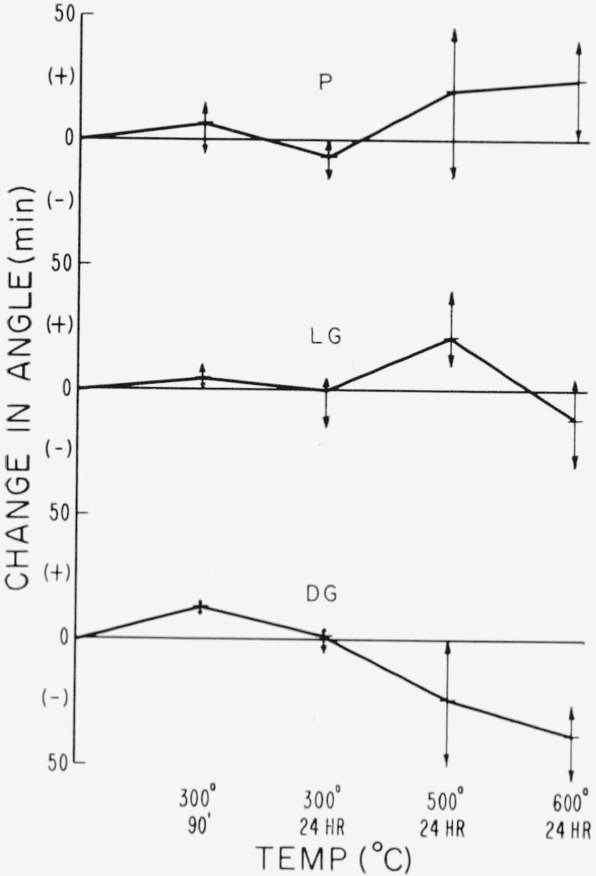
Behavior of apparent optic angle with heat schedule for the three spectral types: pink ruby, light green, and dark green.

The manner in which the apparent optic axial angle varies with the severity of thermal treatment as a function of end type can be seen in figure 13. These data were taken from 21 selected crystal sheets appropriately sectioned and distributed for thermal procedures 1 through 3. Of these crystals eight were very pink ruby, seven were dark green, and six were light green. The complete spread in the data is shown for each temperature-time and average values are joined by the line segments.

It is seen (fig. 13) that the low-temperature (300 °C) behavior is essentially the same for all three types in that the angle is increased to a maximum with short exposure but then diminished by longer exposure. On the average light green has the smallest increase in angle, while dark green has the largest when subjected to 300 °C. However, the high temperatures of 500 and 600 °C cause changes in angle characteristic of each type. Pink ruby and dark green experience changes in angle opposite in sign. The changes in light green show some characteristics of each of the other two.

## 3. Conclusions

The differentiation of muscovite sheet according to the three end-types of very pink ruby, light green, and dark green appears to be substantiated by their response to thermal treatment.

The changes caused by these relatively low-temperature treatments raise a number of questions about the structure of the material. Particular attention is merited by the effects on the visible and the infrared pink associated absorption regions of 0.47 to 0.6 *μ* and 3 to 3.5 *μ*. The 3 to 3.5 *μ* absorption was only found in ruby specimens along with a direct association with the 0.47 to 0.6 *μ* absorption [1]. But thermal treatment can both enhance and bleach out the visible absorption while the infrared absorption is relatively little affected. Too, the visible absorption is a weak structure on the edge of a deep absorption edge. These factors point to a defect structure as being responsible for the 0.47 to 0.6 *μ* absorption, which defect structure is the result of a particular crystal molecular arrangement evidencing the 3 to 3.5 *μ* absorption.

## Footnotes

1 Figures in brackets indicate the literature references at the end of this paper.
